# Potential circulating biomarkers of circulating chemokines CCL5, MIP-1β and HA as for early detection of cirrhosis related to chronic HBV (hepatitis B virus) infection

**DOI:** 10.1186/s12879-019-4130-0

**Published:** 2019-06-14

**Authors:** Liangshan Hu, Yan Zhu, Jingqian Zhang, Wei Chen, Zeyong Li, Lihua Li, Liping Zhang, Donglin Cao

**Affiliations:** 1 Department of Laboratory Medicine, Guangdong Second Provincial General Hospital, No.466 Xingang Middle Road, Haizhu, Guangzhou, 510317 People’s Republic of China; 2 Department of Laboratory Medicine, The First People’s Hospital of Kashgar Prefecture Xinjiang, Kashgar, Xinjiang People’s Republic of China

**Keywords:** CCL5, MIP-1β, Hyaluronic acid, Hepatitis B virus, Cirrhosis

## Abstract

**Background:**

Due to no clinical symptoms in the compensated stage of cirrhosis, it is usually diagnosed when decompensated complications occur. In this study, the noninvasive circulating biomarkers for early detection to compensated stage of cirrhosis in patients with chronic HBV (hepatitis B virus) infection was explored.

**Methods:**

According to the Guideline of Prevention and Treatment of Chronic Hepatitis B (2015 Update), 78 patients with CHB (chronic hepatitis B) were divided into mild group, moderate-to-advanced group, while 73 patients with HBV-related cirrhosis were divided into compensated group and decompensated group. Nineteen cytokines and chemokines, four serum liver fibrosis markers were measured using chemiluminescence. The expression of CCL5 in liver tissue was determined with immunohistochemistry.

**Results:**

The CCL5 expression level in serum increased in CHB patients with aggravated liver injury and significantly decreased in cirrhosis patients with advanced liver fibrosis. ROC analysis revealed that the serum levels of CCL5, HA and MIP-1β were effective in distinguishing patients with cirrhosis from patients with CHB, especially for CCL5. Increasing serum level of CCL5 in CHB patients was severely associated with disease progression.

**Conclusions:**

The serum levels of CCL5, HA and MIP-1β maybe used to distinguish cirrhosis from CHB patients, moreover, CCL5 was the most reliable marker. The increasing serum levels of CCL5 were significantly related to disease progression in CHB patients.

## Background

At first, chronic hepatic injury cause aberrant regeneration of hepatocyte, then formation of fibrous bands induce regenerative nodules in histological development process. Which is named as cirrhosis, then lead to end stage liver disease and portal hypertension [1]. Approximately 1.03 million people of liver cirrhosis die annually worldwide [2]. The chronic infection of HBV (Hepatitis B virus) is the crucial reason of liver cirrhosis or Hepatocellular carcinoma (HCC). The initial stage of HBV-related cirrhosis is asymptomatic, especially compensated cirrhosis stage, and disease progression has already developed to decompensated stage once some complications such as encephalopathy, spontaneous bacterial peritonitis, ascites, and variceal bleeding [2, 3]. The mortality of cirrhotic patients at the decompensated stage without liver transplant is as high as 85% over 5 years. Therefore, early diagnosis in the initial stage of cirrhosis,that is compensated cirrhosis stage, is very essential. In the USA, screening for cirrhosis from patients with chronic hepatitis C had previously been conducted from 1946 to 1965 and remarkable results had been achieved [4]. Screening is also important for patients with hepatic fibrosis or HBV-related cirrhosis in most parts of Asia and Africa.

To diagnose liver fibrosis or cirrhosis, liver biopsy was performed, which was the gold standard. Moreover, to avoid sampling errors, the American Association for the Study of Liver Diseases guidelines recommend obtaining a biopsy with a 16-gauge needle and puncture depth in liver at least 2–3 cm [5, 6]. Patients with asymptomatic chronic HBV related liver disease were reluctant to liver biopsy because of the injury caused by puncture. Conventional imaging examination, such as ultrasonography and CT/MRI show low sensitivity in initial stage of cirrhosis. Noninvasive methods based on “biological” and “physical” approaches are increasingly used in assessing fibrosis in early stage of cirrhosis [7].

Serum C-IV (type IV collagen), PC-III (procollagen III N-terminal propeptide), LN (laminin), HA (hyaluronic acid), collagenases and their inhibitors are considered to be direct markers for the evaluation of liver fibrosis reflecting the turnover of extracellular matrix [8]. The Fibrotest, Forns index, AST-to-platelet ration index (APRL), FIB-4, FibroIndex and ELF are some indices derived from combinations of serum biomarkers to measure fibrosis in patients with chronic hepatitis virus infection (such as B and C) [9, 10].

The inflammation factors including serum cytokines and chemokines are also used to predict prognosis as biomarkers for patients with liver-related diseases, including fibrosis, cirrhosis, and HCC [11–13]. The pro-inflammatory β (C-C) family chemokine members, chemokine (C-C motif) ligand 5 (CCL5) and macrophage inflammatory protein-1 beta (MIP-1β), play an important role in recruiting a variety of leukocytes into inflammatory sites. Previous investigations indicated that CCL5 could mediate hepatic fibrogenesis in murine and human hepatic disease [14–16].

In this work, we attempted to identify which factor could be used as serum biomarkers to predict the progression of CHB or HBV-related cirrhosis through determining 19 inflammatory factors in serum, including CCL5, IL-1β, IL-2, IL-4, IL-5, IL-6, IL-7, IL-8, IL-10, IL-12, IL-13, IL-17, G-CSF (granuclocyte colony-stimulating factor), GM-CSF (granulocyte-macrophage colony-stimulating factor), MCP-1 (monocyte chemoattractant protein-1), IFN-γ (interferon-γ), TNF-α (tumor necrosis factor-α), MIP-1α (macrophage inflammatory protein-1alpha), and MIP-1β, as well as four serum direct liver fibrosis markers including C-IV, PC-III, LN, and HA in CHB patients with (medium-to-severe stage) or without (mild stage) hepatic damage and HBV-related cirrhosis patients at compensated and decompensated phases.

## Methods

### Patient selection

The 73 patients with CHB and 78 patients with HBV-related cirrhosis were studied. All paticipants were positive of HBsAg (hepatitis surface antigen) (≥6 months) and maintained an HBeAg/HBeAb status for at least 3 months. The diagnosis of CHB patients was consistent with the criteria of the Guideline of Prevention and Treatment of Chronic Hepatitis B (2015 Update). To diagnose on cirrhosis that was identified based on clinical diagnostic standards, and supported by radiological diagnosis (CT or MRI) and liver biopsy analysis. Patients co-infected with HCV or HIV (human immunodeficiency virus), besides with liver injury induced by alcohol, cryptogenic cirrhosis, and NAFLD/NASH were excluded. Additionally, patients had undergone therapy with chemoembolization and/or chemotherapy were excluded, too. All patients with CHB were divided into two groups, mild and moderate-to-severe, according to the serum levels of TBIL (total bilirubin) and ALT (alanine aminotransferase) more than two-three times the ULN (upper limit of normal). Patients with TBIL≤85.5umol/L were classified in the mild CHB group, while individuals with TBIL > 85.5umol/L and ALT > 60 IU/L were classified in the moderate-to-severe group. Moreover, patients with cirrhosis were further classified into two groups, compensated group (Child-Pugh A) and decompensated group (Child-Pugh B or C). At the same time, the 52 healthy check-up paticipants as normal healthy control group (NHC), all of which were negtive of HBsAg and normal of TBIL and ALT.

The medical records of the patients were reviewed using the Easylink (LIS) database to obtain gender data, duration of HBsAg-positivity, family history of HBV-infection, the levels of ALT, aspirate aminotransferase (AST), TBIL, albumin(ALB), HBV DNA load, prothrombin time (PT), and α-fetoprotein (AFP). The baseline characteristics of the enrolled patients are summarized (Table 1). This study was approved by the Ethical Committee of Guangdong Second Provincial General Hospital. Informed consents were obtained from all paticipants, and have been stored at our hospital.

**Table 1:** Clinical, histological and pathological characteristics of 52 normal healthy controls, 78 patients with CHB and 73 patients with HBV-related cirrhosis

	NHC	CHB	HBV-related cirrhosis	*P* value
Male	38 (73.1%)	60 (76.9%)	55 (75.3%)	*ns*
Female	14 (26.9%)	18 (23.1%)	18 (24.7%)	*ns*
Age (year)	41.3 ± 10.05	36.0 ± 9.69	47.8 ± 11.6^***^	< 0.001
family HBV-infection history	*no*	12/78	11/73	*ns*
duration of HBsAg positive (year)	*no*	7.6 ± 7.5	11.2 ± 9.1^*^	< 0.05
ALT(U/L)	25.2 ± 6.35	277.3 ± 229.6^###^	166.3 ± 397.0^###***^	< 0.001
AST(U/L)	18.5 ± 3.44	106.2 ± 91.9^###^	110.37 ± 220.1^###^	< 0.001
TBIL(umol/L)	13.4 ± 4.61	48.62 ± 113.12^###^	43.11 ± 85.50^###*^	< 0.001
ALB(g/L)	45.3 ± 9.62	44.6 ± 5.33	37.6 ± 3.85^#*^	< 0.05
PT(s)	12.68 ± 0.68	13.73 ± 1.39	17.25 ± 7.46^##**^	< 0.01
HBV DNA (log_10_ IU/ml, Mean ± SEM)	*no*	5.870 ± 0.194	4.242 ± 0.336^***^	< 0.001
HA(ng/mL)	89.2 ± 26.05	112.3 ± 133.92	228.3 ± 262.9^##**^	< 0.01
LN(ng/mL)	88.6 ± 28.32	77.7 ± 28.68	98.89 ± 58.97	*ns*
PC-III(ng/mL)	76.7 ± 33.56	78.2 ± 55.49	102.9 ± 86.07	*ns*
C-IV(ng/mL)	84.08 ± 21.81	107.19 ± 112.62	120.73 ± 82.93	*ns*

*ns* no significance,^#^: vs NHC *P* < 0.05, ^##^: vs NHC *P* < 0.01, ^###^: vs NHC *P* < 0.001, ^*^: vs CHB *P* < 0.05, ^**^: vs CHB *P* < 0.01, ^***^: vs CHB *P* < 0.001

### Measurement of serum cytokines

The fasting venous blood was drawn, and then serum was separated within one hour and immediately stored at − 70°C until further use. Cytokines and chemokines were measured using a commercially available immunoassay kit (Luminex 200 system; Millipore, Billerica, MA) for IL-1β, IL-2, IL-4, IL-5, IL-6, IL-7, IL-8, IL-10, IL-12, IL-13, IL-17, G-CSF, GM-CSF, IFN-γ, MCP-1, TNF-α, CCL5, MIP-1β and MIP-1α. All samples were determined twice with blinded fashion, means values were used for the analysis.

### Measurement of liver fibrosis markers

The liver fibrosis markers HA, LN, PC-III, and C-IV in serum were measured using clinical diagnostic HA/LN/PC-III/C-IV-enhanced Chemiluminescence kits (Tiguan Biotech, Beijing). Each serum sample with three replicates and 5-point standard samples were performed based on the manufacturer’s instructions, and mean values were used for statistical analysis, too.

### Immunohistochemistry

Paraffin-embedded liver tissue sections were obtained from pathology department of Guangdong Second Provincial General Hospital. Sections were performed antigen retrieval with microwave oven, and then endogenous peroxidase activity was quenched. Non-specific antibody was blocked using normal donkey serum (Jackson ImmunoResearch, Philadelphia, PA, USA). The rabbit anti-CCL5 antibody (1:100 dilution; Abcam, Danvers, MA, USA) was used as primary antibody, and avidin-biotin-horseradish peroxidase was as the second antibody. After that the sections were counterstained with hematoxylin and mounted.

### Statistical analysis

The data were expressed as the means and SD (standard deviation). Comparisons between two groups were performed with unpaired nonparametric Mann-Whitney U test. Moreover, comparisons of more than two groups were analyzed with one-way ANOVA (nonparametric) and multiple comparisons tests. The performance of CCL5, MIP-1β and HA was evaluated based on the calculation of the area under the receiver operator characteristic curve (AUROC), and the cutoff value was calculated using CHB patients as the control. *P*-values below 0.05 for a two-tailed test were considered to be statistically significant. All statistical analyses were performed using GraphPad Prism 7 software for Windows 10.0 (GraphPad Software, Inc.).

## Results

### Baseline patient demographics

The baseline characteristics of the 52 healthy check-up paticipants, the enrolled 78 patients with CHB and 73 patients with HBV-related cirrhosis were shown in Table 1. Among the enrolled patients, more than 75% of patients in both CHB group and HBV-related cirrhosis group were male. The mean age of patients with cirrhosis was older than that of patients with CHB (47.8 ± 11.6 vs. 36.0 ± 9.69), and the average duration of HBsAg-positivity in cirrhosis patients was more than 10 years, and their history of disease was longer than that in patients with CHB (*P* < 0.05). A total of 12 of 78 CHB patients and 11 out of 73 HBV-related cirrhosis patients had a family history of HBV infection, which showed no difference between the two groups. The serum levels of ALT, TBIL, PT, ALB and HBV DNA showed a difference between CHB patients and HBV-related cirrhosis patients (*P* < 0.05) (Table 1).

### Serum cytokines levels in patients with CHB and HBV-related cirrhosis

We also measured the serum levels of 19 inflammatory factors in healthy check-up paticipants, CHB patients and HBV-related cirrhosis patients (Table 2). Compared with healthy check-up paticipants, except for CCL5 and MIP-1β of HBV-related cirrhosis patients, there was no difference for other inflammatory factors of CHB patients and HBV-related cirrhosis patients. Compared with CHB patients, the serum CCL5 level of HBV-related cirrhosis patients was significantly decreased (4375.79 *pg*/mL vs. 11,729.47 *pg*/mL, *P* < 0.001). Consistent with the expression of CCL5, the MIP-1β levels in the serum of cirrhosis patients were slightly lower than that of patients with CHB (49.30 *pg*/mL vs. 75.77 *pg*/mL, *P* < 0.05). No difference was observed between the CHB and HBV-related cirrhosis patients for other inflammatory factors, such as MIP-1α, IL-2, IL-3, IL-5, IL-4, IL-6, IL-7, IL-8, IL-10, IL-1β, IL-12, IL-13, IL-17, G-CSF, GM-CSF, IFN-γ, MCP-1 and TNF-α.

**Table 2:** Cytokine levels in patients with NHC, CHB and HBV-related cirrhosis (pg/mL)

Cytokines	NHC (*n* = 52)	CHB (*n* = 78)	HBV-related cirrhosis (*n* = 73)	*P*-values
CCL5	9335.99 (526.34–18,975.78)	11,729.47 (564.78–15,610.99)	4375.79 (890.92–14,658.46) ^### ***^	< 0.001
MIP-1β	80.45 (29.08–145.58)	75.77 (26.00–225.33)	49.3 (26.56–72.53) ^# *^	< 0.01
MIP-1α	0.31 (0.18–2.15)	0.34 (0.18–3.21)	0.29 (0.18–0.49)	*ns*
IL-2	11.91 (4.32–198.55)	10.86 (5.09–112.64)	12.26 (6.62–271.74)	*ns*
IL-5	2.87 (1.15–8.34)	3.50 (1.50–7.02)	3.18 (1.24–6.08)	*ns*
IL-4	1.11 (0.81–3.88)	1.34 (0.81–5.65)	1.25 (0.89–1.84)	*ns*
IL-6	9.02 (4.08–112.55)	8.80 (4.58–88.19)	9.23 (4.28–162.78)	*ns*
IL-7	1.35 (0.91–8.33)	1.40 (0.98–9.04)	1.29 (0.94–3.25)	*ns*
IL-8	54.47 (8.54–974.35)	47.13 (9.60–3658.40)	27.20 (9.89–254.21)	*ns*
IL-10	4.94 (2.15–98.49)	4.80 (2.30–60.45)	4.10 (2.20–330.67)	*ns*
IL-1β	0.16 (0.09–1.11)	0.15 (0.09–1.69)	0.14 (0.09–0.29)	*ns*
IL-12	4.18 (2.85–158.63)	3.99 (2.55–272.81)	5.63 (2.79–391.44)	*ns*
IL-13	1.18 (0.89–2.44)	1.09 (0.83–2.64)	1.26 (0.91–2.38)	*ns*
IL-17	38.55 (20.63–99.65)	40.92 (20.99–184.84)	37.34 (21.97–93.25)	*ns*
G-CSF	18.88 (13.70–65.23)	17.80 (13.70–71.55)	20.78 (13.70–40.07)	*ns*
GM-CSF	45.26 (15.26–19.48)	46.79 (19.00–280.83)	54.43 (14.54–150.88)	*ns*
IFN-γ	35.12 (20.99–53.54)	33.83 (20.81–58.76)	30.63 (20.81–55.70)	*ns*
MCP-1	20.07 (9.02–189.64)	18.60 (9.02–203.89)	13.97 (9.02–30.28)	*ns*
TNF-α	8.18 (6.99–84.57)	8.11 (6.25–73.31)	8.16 (6.87–96.67)	*ns*

^#^: vs NHC *P* < 0.05, ^###^: vs NHC *P* < 0.001,^*^: vs CHB *P* < 0.05, ^***^: vs CHB *P* < 0.001, *ns* no significance

### Serum CCL5 and MIP-1β in cirrhosis and CHB patients

**Fig. 1:**
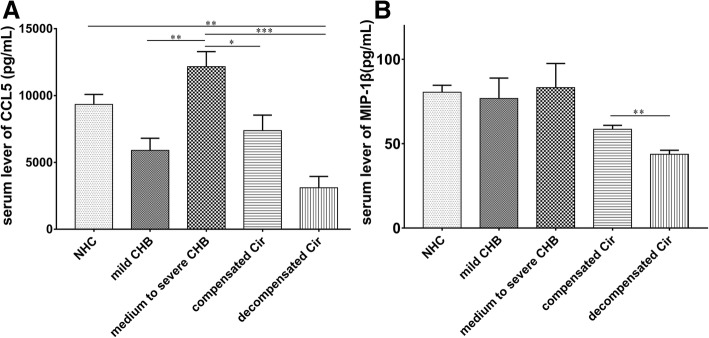
Serum levels of CCL5 and MIP-1β in different phases of CHB and HBV-related cirrhosis patients. **a** Serum levels of CCL5 in CHB patients at mild stage, moderate-to-severe stage, compensated HBV-related cirrhosis and decompensated HBV-related cirrhosis. **b** The serum levels of MIP-1β in CHB patients at mild stage, moderate-to-severe stage, compensated HBV-related cirrhosis and decompensated HBV-related cirrhosis. (*: *P* < 0.05, **: *P* < 0.01, ***: *P* < 0.001)

To further investigate the effect of CCL5 and MIP-1β on pathogenesis of CHB and HBV-related cirrhosis, 78 patients with CHB were divided into mild group and moderate-to-severe group, and 73 patients with HBV-related cirrhosis were divided into compensated group and decompensated group. The results indicated that the serum CCL5 level was dramatically increased in CHB patients with progressive severity from mild to moderate-to-severe stage, but in patients with HBV-related cirrhosis, the opposite trend was observed (Fig. 1a). The MIP-1β level was not different between the two phases in CHB patients. However, the level of MIP-1β was remarkably much lower in patients with decompensated cirrhosis compared to that patients with compensated cirrhosis (Fig. 1b).

### Serum level of liver fibrosis markers

**Fig. 2:**
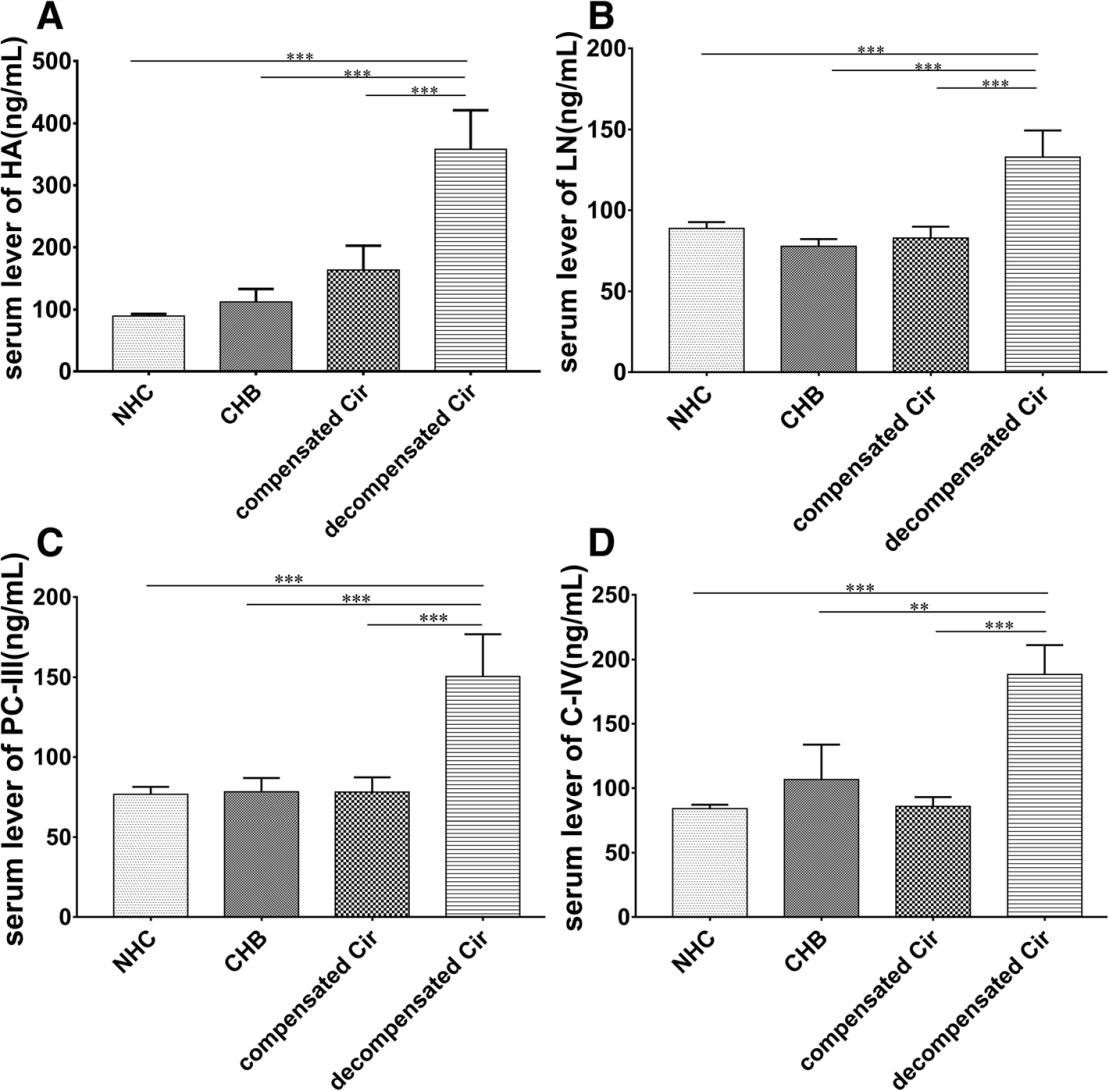
Serum levels of HA, LN, PC-III and C-IV in patients with CHB and HBV-related cirrhosis. Serum levels of HA (**a**), LN (**b**), PC-III (**c**) and C-IV (**d**) in patients with CHB, compensated cirrhosis and decompensated cirrhosis. (**: *P* < 0.01, ***: *P* < 0.001)

The serum levels of HA, LN, PC-III and C-IV were used to assess the degree of hepatic fibrosis in clinical treatment. The results indicated that although the uptrend serum levels of HA, LN, PC-III and C-IV were worsened with disease progression, only the serum HA level of HBV-related liver cirrhosis showed a significant difference compared with healthy check-up paticipants and CHB patients (Table 1). However, significant differences were observed in all four liver fibrosis markers between patients with compensated and decompensated cirrhosis (Fig. 2). The serum levels of HA, LN, PC-III and C-IV continuously increased companied with disease progression, and indicated significant difference in healthy check-up paticipants, the CHB patients and decompensated cirrhosis patients, but there was no difference between the CHB patients and compensated cirrhosis patients.

### ROC curves of serum HA, MIP-1β and CCL5 in patients with CHB and HBV-related cirrhosis

**Fig. 3:**
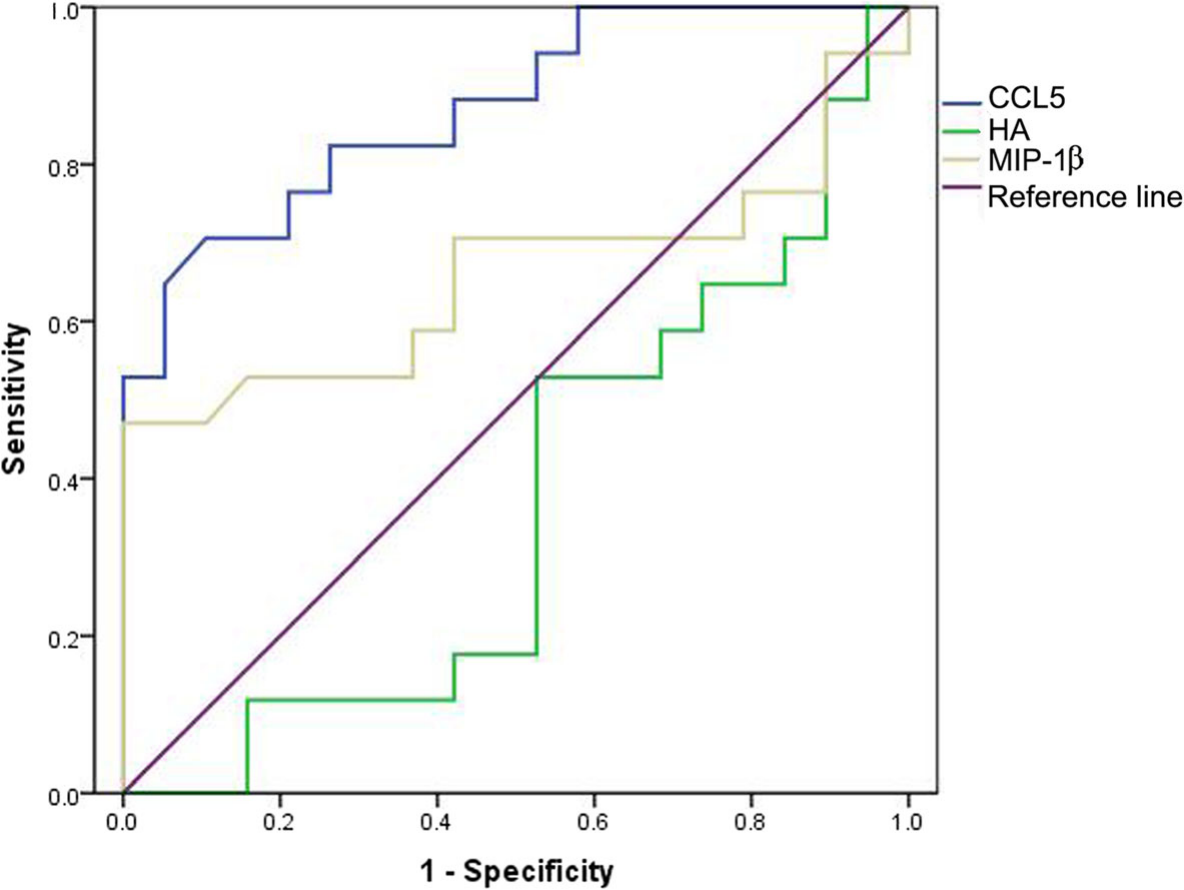
ROC curves of CCL5, HA and MIP-1β to predict patients with CHB (*n* = 78). CCL5: AUC 0.872, 95% confidence interval 0.758–0.985, cutoff value 8569.1, sensitivity 82.4%, specificity 73.7%; HA: AUC -0.632(0.368–1), 95% confidence interval 0.447–0.817, cutoff value 50.18, sensitivity 73.7%, specificity 41.2%; MIP-1β: AUC 0.658; cutoff value 49.75, sensitivity 70.6%, specificity 57.9%

ROC curve analysis of CCL5, HA, and MIP-1β indicated that the serum CCL5 level was the most reliable indicator to predict patients with CHB (AUC: 0.872, − 0.632(0.368–1) and 0.658), although there is a negative correlation between HA and patients with CHB (Fig. 3). Applying 8569.10 *pg*/mL as a cutoff value, 90% CHB patients with moderate-to-severe hepatic damage were distinguished from all CHB patients, while only 30% of patients had high CCL5 levels (> 8569.10 *pg*/mL) in mild CHB patients without hepatic damage, suggesting that elevated serum CCL5 level in CHB patients implies the occurrence of hepatic injury. HA and MIP-1β were another effective factors to evaluate hepatic damage in CHB patients, but the accuracy was less than CCL5.

**Fig. 4:**
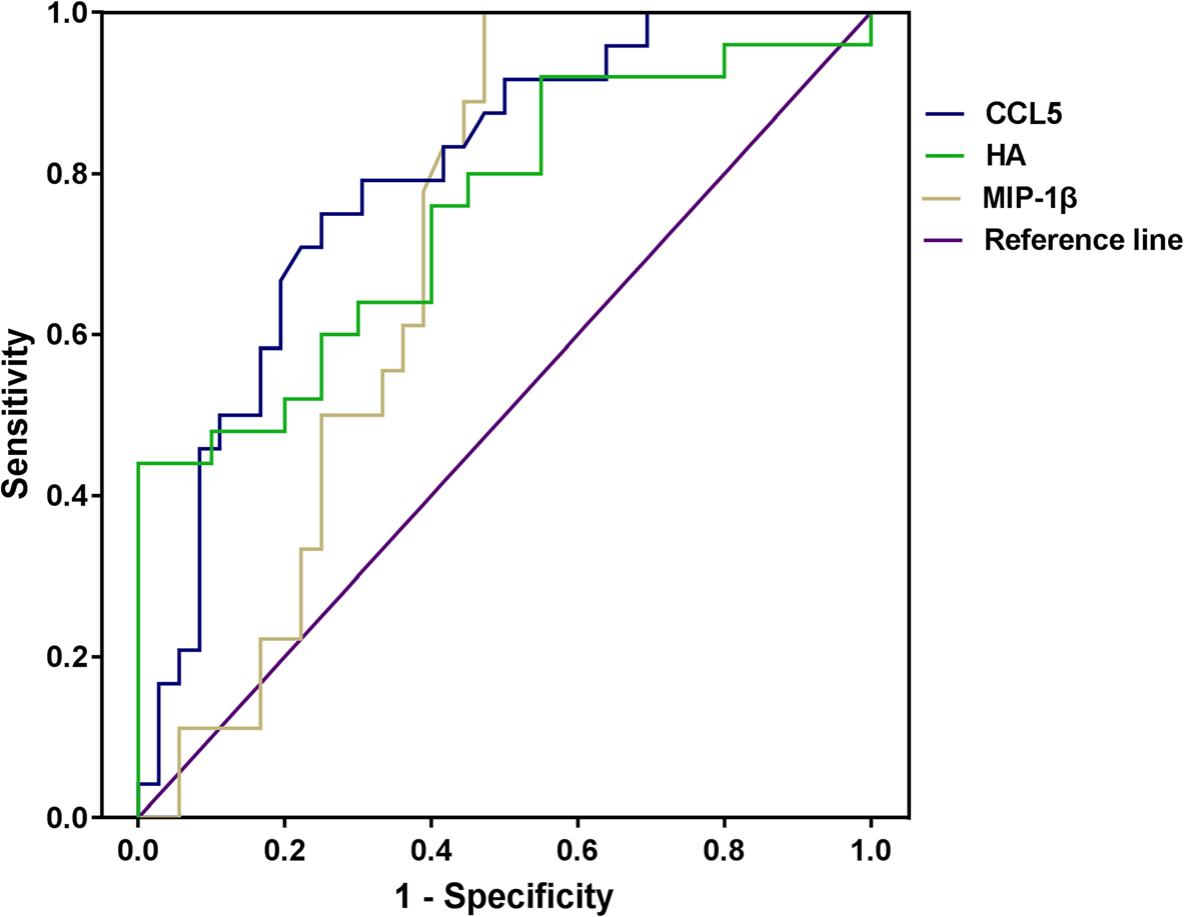
ROC curves for CCL5, HA and MIP-1β for cirrhosis prediction (CHB vs. Cirrhosis). CCL5: AUC 0.8011, 95% confidence interval 0.690–0.913, *P*-value < 0.0001; HA: AUC 0.752, 95% confidence interval 0.611–0.893, *P*-value 0.004; MIP-1β: AUC 0.706, 95% confidence interval 0.571–0.841, *P*-value 0.0143

Considering CHB patients as a control, area under ROC curve (AUROC) of HA, MIP-1β and CCL5 demonstrated that all three factors were reliable in distinguishing cirrhosis from CHB patients, the serum CCL5 level (AUC 0.8011, 95% confidence interval 0.690–0.913, *P*-value < 0.0001) was the most accurate indicator, and HA (AUC 0.752, 95% confidence interval 0.611–0.893, *P*-value 0.004) and MIP-1β (AUC 0.706, 95% confidence interval 0.571–0.841, *P*-value 0.0143) were also used to assess cirrhosis from CHB patients (Fig. 4).

**Fig. 5:**
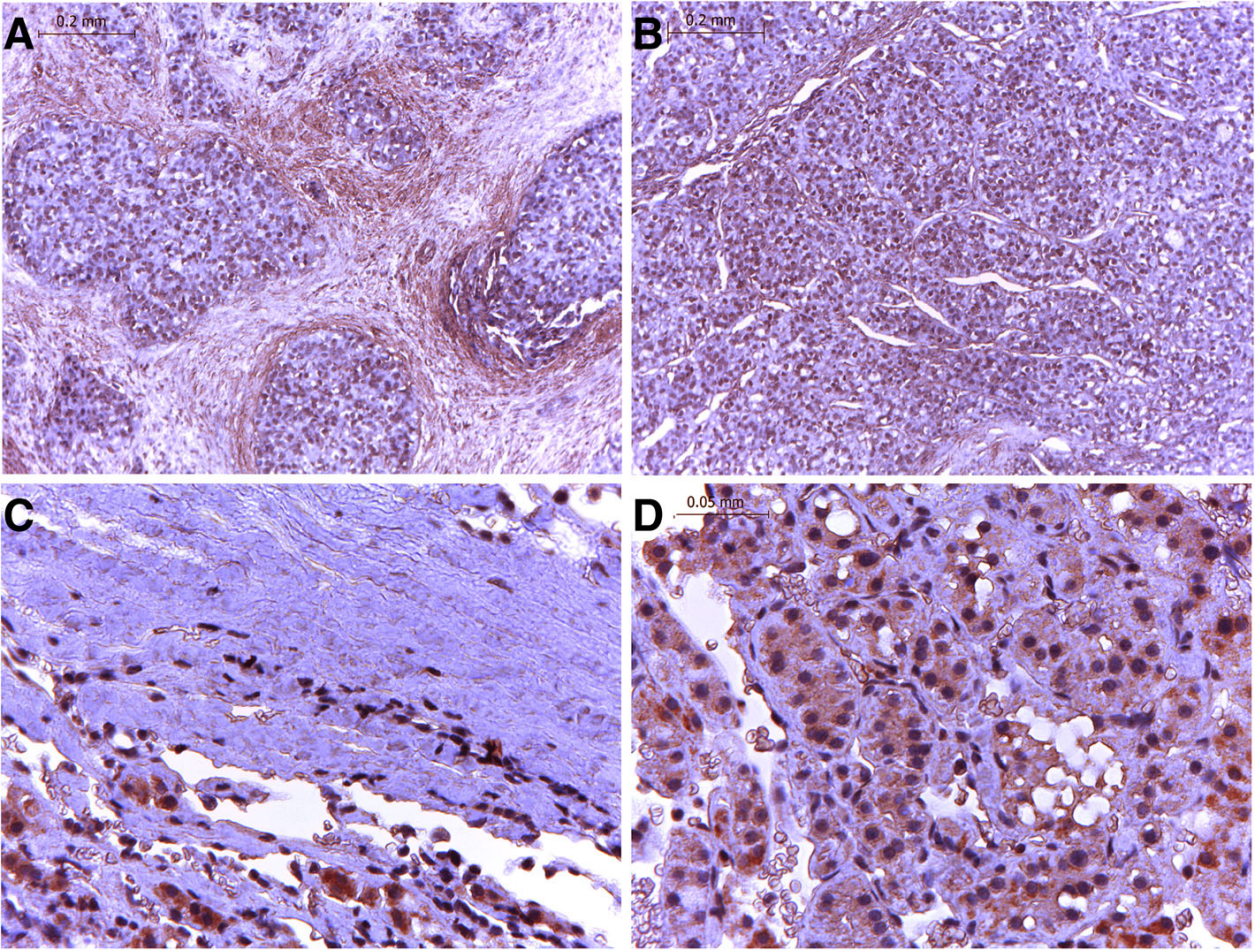
Expression of CCL5 in liver tissue of patients with CHB and cirrhosis. **a**, **c** Cirrhosis, (**b**) (**d**) HBV, (**a**) (**b**) ori × 100, (**c**) (**d**) ori × 400

The hepatic tissue histological structure showed clear, integrated hepatic lobules and cord-like hepatocytes with orderly permutation in HBV liver slices (Fig. 5b). However, in cirrhosis liver tissue, the ordered structure of hepatic lobules was destroyed, and regenerative nodules surrounded with fibrous bands were observed (Fig. 5a). The CCL5 expression level in liver tissue, according to immunochemistry (ICH) analysis, was decreased in the fibrotic liver tissue of cirrhosis patients compared to that of CHB patients. In tissue slices of CHB patients, CCL5 was distributed throughout the cytoplasm near the nucleus (Fig. 5c), while there was not CCL5-positive signals that were observed in destroyed hepatocytes and the fibrous bands of cirrhosis liver slices (Fig. 5c). Similar results for CCL5 expression in hepatic tissue and serum suggest that serum CCL5 levels can indeed reflect the expression of CCL5 in hepatocytes during HBV-related liver disease progression.

## Discussion

The progressive deposit of hepatic fibrosis accompanies chronic hepatic damage resulting from HBV infection, and leading to cirrhosis [2]. Since liver biopsy often leads to severe liver mechanical damage [5, 6], noninvasive evaluation of liver fibrosis has received increasing attention in clinical diagnosis [17–19]. The serum markers of fibrosis and inflammation are two types of indicators and considered for utilization as biomarkers to evaluate liver fibrosis [7, 20–22]. Although many previous studies have attempted to identify accurate or reliable noninvasive biomarkers in predicting liver fibrosis, so far, the results have not been satisfactory.

In the present study, we established a reliable serum indicator from 19 inflammation factors and four serum markers of fibrosis (HA, LN, PC-III, and C-IV) to discriminate patients with significant liver damage or fibrosis in patients with HBV-related CHB and cirrhosis. We observed that only CCL5, MIP-1β, and HA among 23 candidate factors showed significant differences between patients with CHB and HBV-related cirrhosis. To further analyze the reliability of these six factors (CCL5, MIP-1β, HA, LN, PC-III and C-IV) within and between the two groups, CHB and HBV-related cirrhosis group, CHB patients were divided into mild (no detectable liver damage) and moderate-to-severe (abnormal liver function) CHB group. Furthermore, patients with HBV-related cirrhosis were divided into compensated and decompensated group. ROC analysis demonstrated that CCL5 was the most accurate predictor of hepatic injury within CHB patients, and the most reliable indicator of deterioration in patients within HBV-related cirrhosis patients was HA.

HBV infection does not directly result in cytopathic effects for infected hepatocytes, and the host humoral response plays a critical role in the liver damage of CHB patients. Persistent inflammation accelerates hepatocyte injury, which leads to cirrhosis and HCC. Many studies have suggested that various cytokines, such as IL-2, IL-6, IL-8, IL-10, and IL-12 were involved in the progression of HBV-related liver disease and HCC [11, 12, 23–25]. However, in the present study, there was no difference in the serum levels of the tested cytokines in patients with CHB and HBV-related cirrhosis, and only two chemokines CCL5 and MIP-1β among 19 inflammation factors showed significant differences between the two groups. These data showed that in CHB patients, the serum levels of CCL5 were significantly higher in patients with moderate-to-severe CHB than in patients with mild CHB. This result suggested that HBV infection in the early stages of CHB activated host innate and adaptive immunity systems, and T lymphocytes, macrophages, platelets and other immune cells, which produce CCL5 [26], showed sharp proliferation. Therefore, increased serum CCL5 was an unfavorable marker in CHB patients.

In patients with HBV-related cirrhosis, the serum level of CCL5 showed an opposite trend, suggesting that decreased CCL5 level was associated with more severe hepatic damage or worsening progression. This finding reflected the fact that severe hepatic fibrosis of patients with HBV-related cirrhosis in the decompensated phase leads to hepatocyte necrosis, the structural disorder of liver lobules, and the decreased amount of immune cells in liver tissue, which was followed by reduced CCL5 secretion. The average serum CCL5 level of patients with HBV-related cirrhosis was significantly lower than that in CHB patients, it suggested that the serum level of CCL5 was an unfavorable marker of HBV-related liver disease progression. These results are consistent with previous studies obtained in nonalcoholic fatty liver disease, which showed that CCL5 was associated with early stage liver fibrosis progression [26]. CCL5 is a target gene of NF-*κ*B, associated with cancer progression and metastasis [27, 28]. Antagonizing the interaction of CCL5 and its receptor CCR5 with Met-CCL5 could obviously ameliorate the progression of hepatic fibrosis [29] and promote the regression of hepatic fibrosis [15]. MIP-1α, MIP-1β and CCL5 belong to the same C-C chemokine family [27], and the results of MIP-1β were similar to those of CCL5, but the MIP-1α level showed no difference in patients with CHB and HBV-related cirrhosis.

Noninvasive markers of fibrosis are increasingly used in clinical diagnosis, as these factors provide more information at the extreme ends of the liver fibrosis spectrum, ranging from little or no fibrosis to severe cirrhosis [2]. Many serum markers for the noninvasive diagnosis of liver fibrosis have previously been studied to assess hepatic fibrosis [10, 20]. The data obtained in the present study indicated that the serum HA level was significantly elevated, while disease progression in these patients developed from CHB to cirrhosis and the serum levels of three other serum fibrosis markers, LN, PC-III, and C-IV, did not show any difference between patients with CHB and HBV-related cirrhosis. Further analysis indicated that the serum levels of HA, LN, PC-III and C-IV in patients with decompensated cirrhosis were obviously higher than in compensated patients with CHB, but there was no difference in the serum levels of these four markers between patients with CHB and compensated cirrhosis. This finding suggested that severe fibrosis did not occur at the early stage of cirrhosis (compensated phase), whereas the serum levels of HA, LN, PC-III and C-IV sharply increased within clinical decompensated stage. Therefore, the elevation of the serum levels of HA, LN, PC-III and C-IV, particularly HA, is not a favorable marker in HBV-related liver disease.

The ROC curve showed that CCL5 was the most reliable serum marker, and HA and MIP-1β were another effective factors for predicting the aggravation of disease progression in patients with HBV. The level of CCL5 was highly sensitive because the CCL5 serum level increased during liver damage in CHB patients. Taking 8569.10 *pg*/mL as the CCL5 cutoff value, 90% of moderate-to-severe patients with CHB were discriminated from all CHB patients.

## Conclusions

The serum levels of CCL5, HA and MIP-1β were effective in distinguishing patients with cirrhosis from CHB, and among these factors, CCL5 was the most reliable marker. The increasing serum levels of CCL5 in patients with CHB and HBV-related cirrhosis were significantly associated with disease progression.

## Data Availability

The datasets used and/or analysed during the current study available from the corresponding author on reasonable request.

## Abbreviations

ALT: Alanine aminotransferase
APRL: AST-to-platelet ration index
AUROC: Area under ROC curve
CCL5: Chemokine (C-C motif) ligand 5
CHB: Chronic hepatitis B
G-CSF: Granuclocyte colony-stimulating factor
GM-CSF: Granulocyte-macrophage colony-stimulating factor
HA: Hyaluronic acid
HbsAg: Hepatitis surface antigen
HBV: Hepatitis B virus
HCC: Hepatocellular carcinoma
IFN-γ: Interferon-γ
LN: Laminin
MCP-1: Monocyte chemoattractant protein-1
MIP-1α: Macrophage inflammatory protein-1alpha
MIP-1β: Macrophage inflammatory protein-1 beta
PC-III: Procollagen III N-terminal propeptide
Serum C-IV: Type IV collagen
TBIL: Total bilirubin
TNF-α: Tumor necrosis factor-α
